# Impact of Recombined Milk Systems on Gastrointestinal Fate of Curcumin Nanoemulsion

**DOI:** 10.3389/fnut.2022.890876

**Published:** 2022-06-23

**Authors:** Haroon Jamshaid Qazi, Aiqian Ye, Alejandra Acevedo-Fani, Harjinder Singh

**Affiliations:** ^1^Riddet Institute, Massey University, Palmerston North, New Zealand; ^2^Department of Food Science and Human Nutrition, University of Veterinary and Animal Sciences, Lahore, Pakistan

**Keywords:** skim milk powder, heat treatment, curcumin nanoemulsion, digestion, bioaccessibility

## Abstract

Milk powder is an important ingredient in various foods and pediatric formulations. The textural and digestion properties of the formulations depend on the preheat treatment of the milk powder during manufacture. Thus, it is interesting to know how these modifications can influence on the release of fortified bioactive compounds during digestion with a milk matrix. In this study, a curcumin nanoemulsion was incorporated into milks reconstituted from low-heat, medium-heat and high-heat skim milk powders (SMPs) and the milks were subjected to semi dynamic *in vitro* digestion. All the recombined milk systems formed a curd under gastric conditions, which reduced the gastric emptying of protein and curcumin-loaded oil droplets. Because of the formation of heat-induced casein/whey protein complexes, the open fragmented curd formed by the high-heat-treated reconstituted powder resulted in higher protein and oil droplets emptying to the intestine and higher curcumin bioaccessibility. This study provides useful information for how protein ingredients can govern the fate of added health-promoting compounds during digestion.

## Introduction

Defatted dry milk, also known as skim milk powder (SMP), is a major trade item in the food industry because of its ease of transportation, handling and processing, its long shelf life (18–24 months) and its use in a wide range of products (1–3). The manufacture of SMP involves the following steps: fat separation, preheat treatment, concentration and spray drying. The desirable functional properties, such as flavor, color, gelling, foaming etc., that are required for different food formulations are largely dependent on the state of the milk proteins.

Milk proteins are highly influenced by the nature of the preheat treatment applied during powder manufacture and, based on the extent of the heat treatment, SMPs are commonly classified as low heat (MLH), medium heat (MMH) or high heat (MHH) (4). The typical preheat treatments that are used to produce MLH, MMH, and MHH are 70°C/15 s, 85–105°C/60–30 s and 90–120°C/1–2 min, respectively. This preheating step denatures the whey proteins, i.e., β-lactoglobulin (β-LG) and α-lactalbumin (α-LA), forming aggregates that further associate with the casein micelles, and specifically with κ-casein (3, 5). These modified casein micelle/denatured whey protein complexes largely determine the functionalities and applications of SMPs. For example, MLH is preferred for recombined pasteurized milk and cheese making, MMH is preferred for ice cream and chocolate confectionery and MHH is preferred for bakery products and sweetened condensed milk (1, 4, 6).

Recent studies have shown that the heat treatment not only leads to changes in the physicochemical properties of milk but also may have a far greater impact on the gastrointestinal digestion behavior (7–9). Our previous *in vitro* study using a human gastric simulator (HGS) showed that milk without any heat treatment, i.e., raw/unheated milk, formed a close-knit dense clot in the stomach. In contrast, the curds formed by heated and UHT milks were fragmented and crumbled in appearance, with large voids (10). Similar behavior of these liquid milks was also observed in an *in vivo* study in rats (9).

Milk-based formulations have been the most preferred delivery vehicles for vitamin and mineral fortification and the addition of lipophilic drugs and bioactive compounds to promote health (11–13). Among various bioactive compounds curcumin is well-known for its health benefits. However, due to its limited water solubility, degradation and poor bioavailability, its application has been difficult (14, 15). As a result, numerous studies are focusing on employing lipid-based nanosystems to encapsulate curcumin, particularly nanoemulsions, to overcome these constraints (16–18). However, in the nanoemulsion-based milk formulation, the physicochemical interactions of nanoemulsion with milk proteins during processing and digestion within the gastrointestinal tract play an important role in the bioavailability of fortified bioactive compound (19).

Our recent study investigated the *in vitro* digestion of acid and rennet gels that were fortified with curcumin nanoemulsion (17). Despite the fact that these gels had similar rheological and compositional profiles, but their disintegration behavior during dynamic gastric digestion showed a significant impact on the gastric emptying of the oil droplets and, as a result, on the bioaccessibility of the associated lipophilic curcumin during the intestinal phase (17). Similarly, in another study, Niu et al. (12) showed that a high-protein beverage as a food system enhanced the absorption of an enriched coenzyme-Q10-loaded nanoemulsion by increasing the lipolytic activity compared with a coenzyme Q10 nanoemulsion and coenzyme Q10 dissolved in oil. Thus, the nature of the restructuring that occurs in the stomach and the potential interaction of nanoemulsified bioactive compounds with natural food materials have a significant impact on the composition of the food chyme that exits the stomach at different times.

Building on previous work done on heated milk systems, this study sought to evaluate the gastrointestinal digestion profile of recombined milk from milk powders enriched with curcumin nanoemulsion. Different SMPs, i.e., MLH, MMH, and MHH, were reconstituted with a curcumin nanoemulsion and water to achieve an equivalent fat-to-protein ratio. The aim of this study was to evaluate the impact of physicochemical changes in the morphology of the emptied gastric digesta and the clot produced using the dynamic HGS on lipolysis and the release of curcumin during intestinal transit. This research will further our knowledge of the digestion and release of lipophilic bioactive compounds from differently processed recombined milks, as well as the development of dietary food supplements produced from these milk powders.

## Materials and Methods

### Chemicals and Ingredients

MLH, MMH, and MHH SMPs (whey protein nitrogen indices of ≥ 6, 3.3, and 0.3, respectively) and sodium caseinate were purchased from Fonterra Co-operative Group, Auckland, New Zealand. Curcumin (purity ≥ 95%) was purchased from Xi’an Lukee Bio-Tech Co. Ltd, Xi’an, China. Soybean oil (Essenté) was purchased from Davis Trading Company, Palmerston North, New Zealand, and was used without further purification. The following chemicals were purchased from Sigma-Aldrich (St. Louis, MO, United States): pepsin from porcine gastric mucosa (EC 3.4.23.1; product no. P7125), pancreatin (EC 232.468.9; P7545) from porcine pancreas (8 × USP specifications) and bile bovine (EC 232.369.0; B3883). All other chemical reagents and solvents were of analytical grade. Milli-Q water (Millipore Corp., Bedford, MA, United States) was used to prepare all solutions.

### Preparation of Recombined Milk Systems Loaded With Curcumin Nanoemulsion

A curcumin-loaded oil-in-water nanoemulsion, with an average size of 187 ± 11 nm and containing 20% soybean oil, 0.03% curcumin and sodium caseinate as an emulsifier, was manufactured according to a previous method (17). Briefly, both oil and aqueous phases were homogenized (Ultra-Turrax) for 2 min at ambient temperature, which was further passed through double-stage high-pressure homogenizer (APV 2000, Albertslund, Denmark) for 4 cycles at 350/50 bar pressure, to obtain fine nanoemulsion. This curcumin nanoemulsion was further mixed with the SMPs, i.e., MLH, MMH, and MHH, followed by dilution with Milli-Q water to achieve final protein and oil contents of 3.7 and 5%, respectively. The final solutions were then stirred for 2 h at room temperature to allow complete dissolution and were immediately stored at 4°C overnight before being subjected to digestion.

### Dynamic *in vitro* Gastric Digestion

A dynamic gastric model—the HGS designed by Kong and Singh (20)—was used for the *in vitro* gastric digestion of all milk samples. The method described in Qazi et al. (17) was used in the present study with a slight modification. Briefly, 200 g of prewarmed milk sample at 37°C was mixed with 31.3 mL of simulated salivary fluid (SSF) and kept in a water bath for 2 min at 37°C. Before gastric digestion, 28 mL of simulated gastric fluid (SGF) containing pepsin was mixed with the sample to mimic the fasting state of the human stomach. After the food mix had been transferred into the latex stomach chamber, the titration pumps that dosed the SGF and the pepsin separately at a controlled rate of 2.5 mL/min (2.0 mL/min of the 1.25× concentrated SGF and 0.5 mL/min of pepsin solution) were switched on (9). The rollers installed in the HGS contracted three times per minute to simulate the actual peristaltic contraction of the stomach. A 50 mL sample of gastric digesta was emptied after every 20 min and, to mimic human gastric sieving, the emptied gastric digesta was passed through a stainless-steel sieve (pore size approximately 1 mm). The maximum digestion time was 240 min; however, to analysis the changes in the structure of the curd within the stomach and in the emptied gastric digesta, the whole digestion process was terminated at selected timepoints, i.e., 20, 60, 120, 180, and 240 min. Gastric curds collected at these timepoints were freeze-dried and pulverized into powder for further investigation. The pH of the gastric digesta emptied at every 20-min interval was immediately recorded and was assumed to be similar to the pH within the HGS. Further, to stop the activity of pepsin, the pH of the digesta samples was adjusted to 7 by 1 M NaOH and/or 1 M HCl and were stored at 4°C for further compositional analysis.

### Physicochemical Analyses of Emptied Digesta and Gastric Clot

The emptied digesta samples collected at selected timepoints were further chemically analyzed for dry matter and oil content according to the method described in the previous study (17). Similarly, to analyze the changes in the weight of the curd, the gastric digestion was terminated at 20, 60, 120, 180, and 240 min for all recombined milk systems. The contents of the HGS were collected and passed through the 1-mm sieve to separate the solid curd and the liquid gastric digesta. The clot was weighed immediately before being subjected to microscopy to analyze microstructural changes.

### Protein Profile of Gastric Clot and Emptied Digesta

The time-dependent hydrolysis of the proteins in the initial and digested samples (gastric curd and emptied digesta) of MLH, MMH, and MHH in the HGS was determined by analyzing the protein composition of the samples as a function of the digestion time using sodium dodecyl sulfate polyacrylamide gel electrophoresis (SDS-PAGE) as described in Qazi et al. (17). Briefly, initial and emptied digesta samples from selected timepoints were diluted five times with Milli-Q water. These samples were then mixed with sample buffer at a ratio 1:1, and 7 μL of each mixture was loaded into each well. For solid curd samples, 4.5 mg of freeze-dried sample was mixed with sample buffer and 10 μL was loaded into each well. The electrophoresis analysis was conducted at 120 V for approximately 90 min. After staining and destaining, these gels were scanned using a Molecular Imager Gel Doc XR system (Bio-Rad Laboratories, Hercules, CA, United States).

### Microstructure of Curds and Gastric Digesta

The microstructural changes in the milk systems, i.e., MLH, MMH, and MHH, were observed using a confocal laser scanning microscope (Leica SP5 DM6000B; Leica Microsystems, Heidelberg, Germany). A 50 μL aliquot of Nile Red [0.1% (w/v), acetone-dissolved] and a 50 μL aliquot of Fast Green (1.0%, dissolved in water) were added into 500 μL of sample (original and gastric digesta samples) to stain the lipid and protein, respectively. However, the solid curd samples were stained by submerging them into both dyes for 10 min to facilitate the diffusion of the dyes into the samples. An appropriate amount of stained sample was placed on the concave surface of a confocal microscope slide (Sail; Sailing Medical-Lab Industries Co., Ltd, Suzhou, China), covered with a coverslip and examined under a × 63 oil immersion lens. An argon laser with an excitation wavelength at 488 nm was used for Nile Red and a He–Ne laser with an excitation wavelength at 633 nm was used for Fast Green. The images of the oil and protein microstructures were obtained directly from the supporting microscope software (Leica) and were stored with 1,024 × 1,024 pixel resolution.

### *In vitro* Intestinal Digestion

The gastric digesta emptied at 20, 120, and 240 min were further submitted to *in vitro* intestinal digestion. The INFOGEST *in vitro* digestion technique was used to imitate *in vitro* intestinal digestion under static conditions (21). To reach a final ratio of 1:1, 20 mL of simulated intestinal fluid containing 10 mM bile and pancreatin (trypsin activity 100 U/mL) was mixed with a gastric digestion sample in a digestion flask. The digestion was carried out at 37°C for 2 h, and the pH of the mixture was constantly checked and corrected to 7 using 0.1 M and 1 M NaOH.

The release of FFAs during lipid digestion was detected using a pH-stat technique and calculated using the following equation:


$$\frac{\mu {\text{mol}}_{fattyacid}}{mL_{gastricdigesta}}=\frac{\left[V_{NaOH}\left(t\right)-V_{NaOH}\left(a\right)\right]-C_{NaOH}\times 1,000}{V_{gastricdigesta}}$$

Here, *V*_*NaOH*_ (*t*) is the volume of NaOH used at neutralize total acid released at digestion time *t*, *V*_*NaOH*_ (*a*) is the volume of NaOH used to neutralize acid released as amino acids during digestion time *t* μmol, *C*_*NaOH*_ is the concentration of the NaOH solution used to titrate the acid released in 2 h, i.e., 0.05 M, and *V*_*gastric digesta*_ is the volume of the gastric digesta, i.e., 20 mL.

### Particle and Oil Droplet Sizes of Gastrointestinal Digesta

The mean particle sizes of the gastrointestinal digesta after collection were determined using a Mastersizer 2000 (Malvern Instruments Ltd, Malvern, Worcestershire, United Kingdom). The powders were diluted in water to achieve a saturation between 14 and 16% (concentration of ∼0.001%). An emptied digesta sample at a selected timepoint was immediately added to an automated small volume sample dispersion unit (Hydro2000S) prefilled with distilled water until an obscuration between 10 and 15% had been reached. Similarly, the impact of the gastric digestion on the oil droplet size was analyzed by mixing the emptied gastric digesta sample with a mixture of 2% SDS and 50 mM EDTA at a ratio of 1:2. The mixture was then gently mixed for an hour to dissociate the clusters of protein stabilizing the oil droplets. This dissolved mixture was then used to measure the oil droplet size using polydisperse analysis and the droplet size was recorded as the surface-weighted diameter (*D*_3,2_). All measurements were conducted at room temperature and the average particle and droplet diameters of the emptied gastric digesta were measured in triplicate.

### Curcumin Bioaccessibility

After the small intestinal phase, the samples were separated into two portions: a micelle sample and a total digest sample. To isolate the mixed micelle fraction containing solubilized curcumin, a 20 mL aliquot of the entire digesta was centrifuged (38,200 *g* and 20°C for 30 min) using a T-865 rotor (Sorvall WX Ultra 100; Thermo Scientific, Asheville, NC, United States) and the clear supernatant was collected. To separate hydrophobic curcumin, the mixed micelle fraction and the whole digest fraction were dispersed in chloroform, vortexed and centrifuged for 60 min at 3,800 *g*. The following equation was used to calculate the bioaccessibility of curcumin:


$$Bioaccessibility\left(\%\right)=100\times \frac{C_{micelle}}{C_{digesta}}$$

In this formula, the measured curcumin concentration in the micelle phase is represented by *C*_*micelle*_, and the actual curcumin concentration in the intestinal digesta is represented by *C*_*digesta*_.

### Statistical Analysis

Data plotting and statistical analysis (one-way analysis of variance and Tukey’s multiple comparison test) were performed using Minitab software (Minitab version 16; Minitab, Inc., State College, PA, United States). The results were expressed as the mean ± standard deviation of at least two replicates. Differences were considered to be statistically significant at a level of *p* < 0.05.

## Results and Discussion

### Gastric Phase

#### Coagulation Behavior of Recombined Milks in the Human Gastric Simulator

**FIGURE 1:**
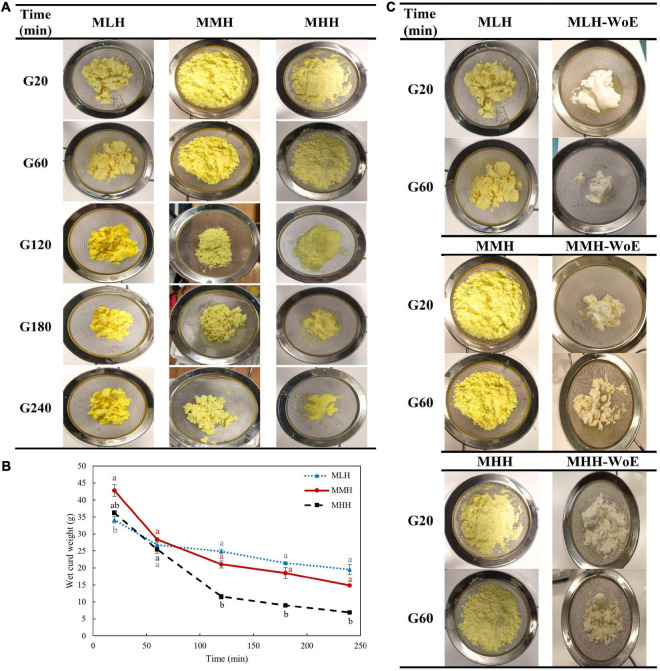
**(A)** Images and **(B)** wet weights of the curds formed during the gastric digestion (simulated gastric fluid with pepsin) of 200 g of recombined milks with added curcumin nanoemulsion, i.e., low heat (MLH), medium heat (MMH) and high heat (MHH), at 20, 60, 120, 180, and 240 min of gastric digestion. **(C)** Comparison of the structures of the curds formed with and without curcumin nanoemulsion (WoE) at 20 and 60 min of gastric digestion.

The formation of curd in all three recombined milk systems in the HGS was seen visually; however, the structures of the curds formed during the first 20 min of digestion were different for all three systems (Figure 1A). The curd formed by MLH at 20 min was fragmented and had a crumbled texture. This curd appeared to be similar to the curd formed during the gastric digestion of pasteurized milk (22). The curds formed by MMH and MHH were soft with loose structures. The differences among the nature of the clots formed may have been due to the degree of preheat treatment applied to these milk systems before spray drying. The high heat treatment of milk results in greater denaturation of the whey proteins and their interactions with the casein micelles, which then results in the formation of a softer curd during digestion (9).

The size of the curd fragments of MLH reduced gradually with increasing digestion time. After 240 min, these curd pieces were less integrated and separated into smaller pieces. The soft curds formed at 20 min by MMH and MHH, which had similar appearances, transformed into two distinct shaped curds by the end of the gastric phase. The amount of MHH curd decreased markedly and the curd formed gave the appearance of breadcrumbs that shrunk to a smaller size at longer digestion times. This curd was similar to the images of the curd obtained during the dynamic *in vitro* gastric digestion of sheep milk that was homogenized and heated to 95°C (23). However, for MMH, the initial soft curd transformed into numerous compact curd fragments over time (Figure 1A). The differences in these curd structures may have been due to the level of whey protein associated with the casein micelles (3, 9).

The weight of the curd formed inside the HGS was also recorded after the curd had been passed through a 1-mm sieve. MHH disintegrated more rapidly in the gastric chamber than MMH and MLH (Figure 1B), which is consistent with previous work demonstrating decreased curd retention in the stomach with an increase in the amount of heat applied to the milk (9, 24).

To determine the influence of the curcumin nanoemulsion on the structure of the curds formed in the stomach, all recombined milks without the addition of nanoemulsion (i.e., reconstituted skim milks) were subjected to gastric digestion for 1 h. The reconstituted skim milks formed compact structured clots at both 20 and 60 min of digestion; they were significantly different from the clots containing curcumin nanoemulsion (Figure 1C). This may have been due to the entrapment of a large number of emulsified curcumin-loaded oil droplets within the curds, which prevented close contact among the coagulating casein particles. A previous study found that the homogenization of whole milk resulted in the coating of the fat globules with casein/whey protein, which then became embedded into the coagulum, leading to changes in the structure of the curd (25). Similarly, sodium-caseinate-stabilized nanoemulsion oil droplets may have interacted with the casein/whey protein aggregates, leading to alterations in the structure of the protein curd.

#### Microstructures of Gastric Curd and Emptied Digesta

**FIGURE 2:**
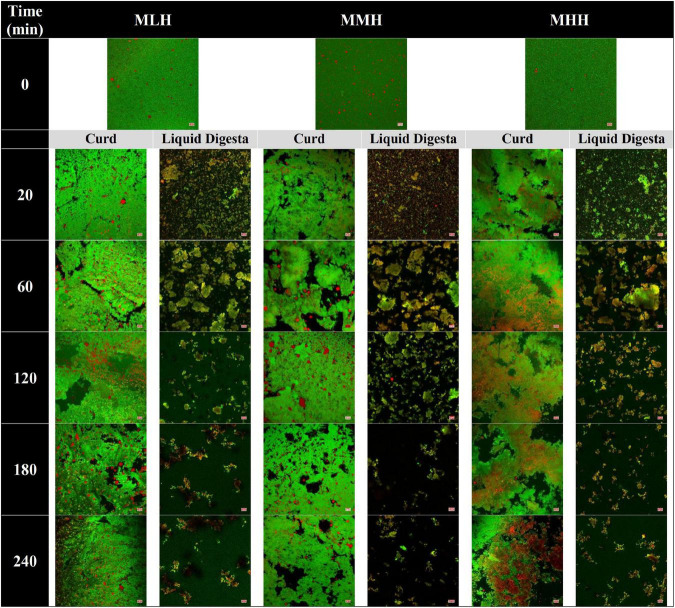
Confocal microscopy images of gastric curd and liquid digesta formed at 0, 20, 60, 120, 180, and 240 min of gastric digestion of low-heat (MLH), medium-heat (MMH), and high-heat (MHH) recombined milk systems. Red shows the oil droplets and green shows the milk proteins. The scale bar corresponds to 10 μm for all micrographs.

**FIGURE 3:**
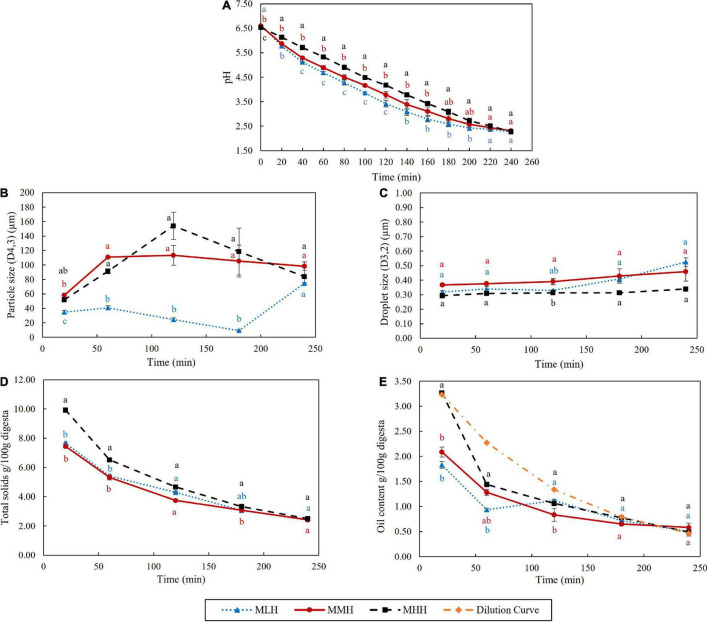
Effect of dynamic *in vitro* gastric digestion of different recombined milk systems on changes in **(A)** pH, **(B)** particle size (*D*_4,3_), **(C)** oil droplet size (*D*_3,2_), **(D)** total solids and **(E)** oil content of the emptied gastric digesta. The standard error is indicated by error bars.

The microstructural variations in the curds formed by the recombined milk systems in the HGS were investigated using a confocal laser scanning microscope (Figure 2, curd). Before digestion, emulsified oil droplets were uniformly distributed in the protein aqueous phase in all three recombined milk systems. At an early stage of digestion (20 min), a close-knit network of protein was observed for MLH and MMH, whereas the curd formed by MHH had a more open network with numerous irregular dark intermittent holes; this structure was similar to that seen in UHT milk during gastric digestion (9). The protein matrix appeared to shrink as the digestion time increased, and the structure of the curds became considerably more open, with blocks of aggregated proteins (Figure 2, 240 min). A portion of the oil droplets in the casein network or the surrounding pores of the casein network appeared to be physically trapped. Previous studies investigating the gastric colloidal behavior of milk proteins in different dairy products have shown that the oil droplet/fat globule size increases with increasing digestion time (9, 22, 26). In our study, because the initial average size of the oil droplets was so small (<200 nm), most of the oil droplets that were embedded within the protein microstructure remained invisible under the microscope. Although the droplet size data showed a small increase in size toward the end of digestion, i.e., approximately 300 nm (Figure 3C), it was not noticeable under the microscope. The more open microstructure of the MHH curd generated by the gastric digestion showed these stained nanosized oil droplets as red zones inside the protein matrix, whereas these zones were less noticeable in the MLH and MMH curds. Conversely, the larger droplets observed in the curd micrographs could represent a limited degree of oil droplet coalescence within the curd matrix.

In contrast, in the stomach environment, the emptied digesta of all three systems revealed a constant disintegration of the associated protein networks (Figure 2, liquid digesta). Initially at 20 min, the digesta samples contained numerous small-sized curd particles with evenly distributed oil droplets. Within the first hour of digestion, these small curd particles grew in size and became flake-like, before gradually shrinking in size until the end of digestion.

#### Physicochemical Changes in Emptied Liquid Digesta

The pHs of the recombined milk samples during gastric digestion were recorded every 20 min for up to 240 min (Figure 3A). The initial pHs of the samples in the stomach (before mixing with the SSF and 28 mL of SGF) were 6.64, 6.58, and 6.54 for MLH, MMH, and MHH, respectively. With the gradual addition of SGF during digestion, the pH of the emptied digesta decreased gradually over time to about pH 2.29 by the end of 240 min. Throughout the 240 min of digestion, MHH had a significantly slower decrease in pH, followed by MMH and MLH. These results are in agreement with those reported by Ye et al. (9), in which UHT milk demonstrated a greater pH buffering capacity than unheated and pasteurized milk during the gastric phase. The higher buffering capacity of the heated milk system was possibly due to changes in the structure and composition of the casein micelles during heat treatment. The different preheat treatments applied during the manufacture of SMPs result in different extents of whey protein denaturation. These denatured whey proteins further interact with κ-casein, forming complexes with κ-casein on the surface of the casein micelles (27). The higher level of whey protein association in MHH resulted in a more fragmented structure of the curd in the stomach (Figure 1A), which could affect the rate of pH decrease during gastric digestion.

The variations in the weight-to-volume diameter (*D*_4,3_) of the emptied digesta during gastric digestion were significantly different in all three recombined milk systems (Figure 3B). The *D*_4,3_ values of the MMH and MHH digesta samples increased rapidly between 20 and 60 min from 58 and 52 μm to 111 and 91 μm, respectively. In the MHH digesta sample, this particle size reached 154 μm at 120 min, before dropping to 84 μm at the end of the digestion. In contrast, there was no discernible change in the particle size of the MMH digesta beyond 60 min of digestion. In comparison with the MMH and MHH digesta, the *D*_4,3_ of the MLH digesta increased slightly to 35 and 41 μm after 20 and 60 min, respectively, before dropping to 9 μm at 180 min. Interestingly, during the last hour of digestion, there was a substantial increase in the particle size, bringing it close to the *D*_4,3_ values of the MMH and MHH digesta. During the gastric digestion, the sizes of the emulsion droplets embedded within the curd particles were observed to be less affected (Figure 3C), which is similar to the behavior observed for curcumin nanoemulsion that were enriched within dairy gels (17). This demonstrated that the nanoemulsion embedded within the curd particles had greater stability to the structural transformations during the gastric phase.

During the first 120 min of gastric digestion, MHH showed a significantly (*p* < 0.05) faster emptying of total solids than MLH and MMH (Figure 3D). These results are in agreement with the gastric curd images (Figure 1A) and the wet weights of the curds (Figure 1B), showing faster disintegration of the MHH samples. The curds formed by the other two recombined milk systems retained the maximum total solids, while allowing a small fraction to pass through to the liquid digesta. This difference became less pronounced as the digestion progressed and, at the end of the process, the total solids contents of the MLH, MMH, and MHH digesta were almost identical.

The oil contents emptied in the gastric digesta at different digestion times from the different recombined milk systems were also analyzed (Figure 3E). The MHH digesta sample emptied at 20 min had significantly higher emptying of oil content, i.e., approximately 3.27%, than the MMH and MLH gastric digesta samples, i.e., approximately 2.09 and 1.83%, respectively. The oil content in the MHH digesta sample at 20 min was similar to the hypothetically calculated oil value (i.e., the dilution curve), based on dilution of the digesta because of the gradual addition of SGF at different timepoints. The more open microstructure of the curd formed by MHH clearly allowed more oil droplets to be released into the digesta compared with the other two recombined milk systems, which entrapped them within the curd. With the progression of digestion, the oil content in the digesta samples of all three recombined milk systems gradually decreased with no significant differences at digestion durations of longer than 120 min. Overall, the oil content of the MHH digesta remained significantly higher than those of the MLH and MMH digesta, indicating that it disintegrated more rapidly in the HGS, as explained in section “Coagulation Behavior of Recombined Milks in the Human Gastric Simulator.”

#### Kinetics of Milk Protein Disintegration During Gastric Phase

**FIGURE 4:**
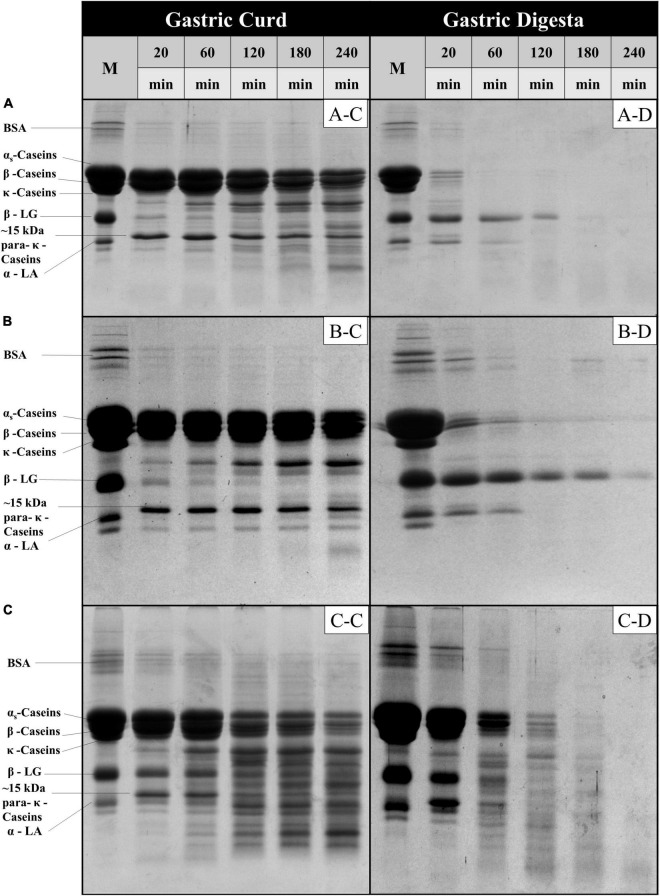
SDS-PAGE patterns (under reducing conditions) of freeze-dried gastric curd (C) and liquid digesta (D) samples obtained at selected timepoints of gastric digestion of MLH **(A)**, MMH **(B)** and MHH **(C)**. M stands for the original samples before digestion and all other samples are labeled appropriately in the figure.

The protein fraction of the gastric curd and emptied digesta samples of all three milk systems were characterized by sodium dodecyl sulfate-polyacrylamide gel electrophoresis (SDS-PAGE) under reducing conditions (Figure 4). Figures 4A-C,B-C,C-C depicts the protein hydrolysis by pepsin in the curd samples recovered from the stomach at selected timepoints. The changes during the digestion were then compared with the protein profile of the native samples (lane M). Overall, MHH had faster protein hydrolysis in the curd than MLH and MMH. With increasing digestion time, the intensities of the α_*s*_-casein and β-casein bands reduced; several peptide bands with varied molecular weights (from 10 to 20 kDa) were clearly evident from 60 min onwards in the MHH curd samples (Figure 4C-C). In contrast, MLH and MMH did not show any notable drop in intensity over time for casein bands other than κ-casein (Figures 4A-C,B-C). The only trend that all three systems had in common was the disappearance of the κ-casein bands during the first 20 min of digestion as a result of pepsin hydrolysis, which led to the formation of a para-κ-casein band with a molecular weight of 15 kDa (10). The β-LG and α-LA bands were more prominent in the MHH curd samples, compared with the other samples, and decreased gradually as the digestion progressed (Figure 4C-C). This confirmed that the whey proteins were involved in the formation of the MHH curd structure because of the association of whey proteins (β-LG and α-LA) with the casein micelles during powder manufacture. For MLH and MMH, very faint whey proteins bands were seen at 20 min of digestion; they then disappeared (Figures 4A-C,B-C).

In contrast, for MLH, MMH, and MHH, the casein bands on the SDS-PAGE gels of the digesta samples disappeared after 20, 60, and 120 min of digestion, respectively (Figures 4A-D,B-D,C-D). Casein was emptied into the gastric digesta in a pattern that was comparable with but opposite to that of the curds retained in the gastric chamber. That is, the greater was the amount of curd that was retained in the stomach, the lesser was the amount of casein that was discharged into the digesta. MHH also had greater peptide release into the digesta. The higher rate of protein digestion in heated milk has been attributed to the loose structure of the curd (9, 25). This creates a larger surface area for pepsin diffusion into the curd, resulting in faster protein breakdown and a higher rate of curd particle (1 mm) emptying into the digesta. The presence of intact β-LG and α-LA in the digesta during the early stages of MLH and MMH digestion was attributed to the fact that β-LG is not hydrolyzed by pepsin in its native state. However, after 60 min of digestion, the α-LA band disappeared, but the β-LG band faded steadily as the digestion progressed. In contrast, these whey protein bands disappeared and peptide bands appeared in the MHH digesta samples after 60 min.

### Intestinal Phase

#### Particle Size

**FIGURE 5:**
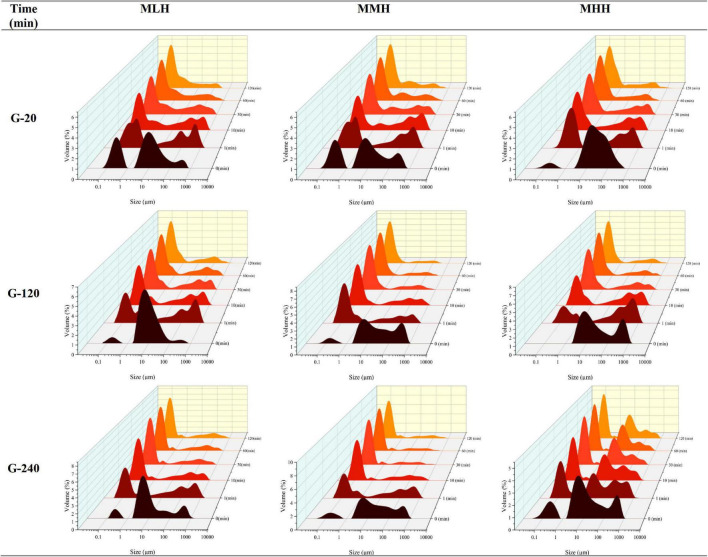
Changes in particle size distribution of emptied gastric digesta (20, 120, and 240 min) before (0 min) and during the intestinal digestion of MLH, MMH, and MHH at different times (1, 10, 30, 60, and 120 min).

The gastric digesta emptied at 20, 120, and 240 min were further subjected to a static *in vitro* intestinal digestion. During the intestinal phase, portions of the digesta were removed at 1, 10, 30, 60, and 120 min and immediately analyzed for changes in particle size distribution (Figure 5). The MLH and MMH gastric digesta emptied at 20, 120, and 240 min had trimodal size distributions whereas the MHH gastric digesta had a bimodal distribution at 20 and 120 min and a trimodal distribution at 240 min. Within 1 min of intestinal digestion, the particle size, initially falling under the peak in the range 1–100 μm, distributed into two distinct peaks: a narrow peak between 0.1 and 1 μm and multimodal peaks between 10 and 1,000 μm. This initial breakdown of particles within the first minute of digestion was consistent across all gastric digesta of the recombined milk systems that were emptied at selected timepoints. As the digestion progressed, the area under the multimodal peaks, representing undigested particles, decreased continuously until 120 min of intestinal digestion, indicating disintegration of the larger curd fragments. In contrast, the unimodal peak, representing small, digested particles, became narrower and the volume of particles with size between 0.1 and 1 μm increased steadily. This continuous increase in newly generated small particles over time can be connected to the mixed micelles generated from the hydrolysis products of lipids and bile salts, which play a key role in enhancing the bioaccessibility of lipophilic bioactive substances (28, 29).

#### FFA Release

**FIGURE 6:**
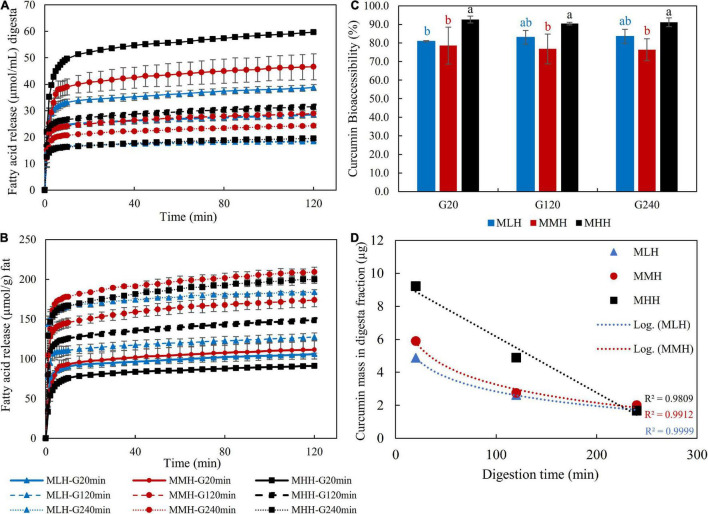
Free fatty acid release profile **(A)** per milliliter of gastric digesta and **(B)** per gram of fat, **(C)** bioaccessibility of curcumin and **(D)** curcumin mass in digesta fractions after *in vitro* gastrointestinal digestion.

Figure 6A shows the FFA release profiles per milliliter of digesta sample emptied after 20, 120, and 240 min of gastric digestion throughout 120 min of intestinal digestion. The FFA concentration reached a plateau during the first 10 min of intestinal digestion, indicating that most of the lipid in the digesta samples had been digested. This behavior has been observed previously in the gastrointestinal digestion of a high-protein beverage incorporating a coenzyme Q10 nanoemulsion system (12) and in dairy gels loaded with curcumin nanoemulsion (17). Beyond 10 min, the moderate and more sustained FFA release with the progression of the intestinal digestion can be associated with multiple factors, i.e., enzyme-to-oil ratio, agglomeration of the digestion products generated as a result of lipolysis at the oil droplet interface and characteristics of the oil, which include their type, droplet size and nature of the emulsifier stabilizing them (17, 26, 30, 31). For all recombined milk systems during the intestinal phase, the gastric digesta emptied at 20 min released significantly more FFAs than the digesta emptied at 120 and 240 min. Moreover, the release of FFAs was significantly (*p* < 0.05) greater in the MHH gastric digesta at 20 and 120 min than in the MLH and MMH digesta at the same timepoints. This followed the same pattern as that of the emptying of the oil content into the digesta, namely G-20 > G-120 > G-240 (Figure 3E), and comparable behaviors were also observed during the gastrointestinal digestion of dairy gels (17).

However, when the amount of FFAs released was calculated per gram of fat as a function of time, the trend changed for all recombined milk systems (Figure 6B). The gastric digesta emptied at 240 min had the highest rate of lipolysis, followed by the gastric digesta emptied at 120 min, and finally the gastric digesta emptied at 20 min in all recombined milk systems. Similar behavior was observed by Guo et al. (26) when they evaluated FFA release during the intestinal digestion of whey protein emulsion gels. They explained these changes as a result of the changes in the gel structure or the colloidal structure of gel fragments during intestinal digestion, which may have impacted the hydrolysis of the oil droplets incorporated within the protein matrix. This appears to be the most likely mechanism regulating lipid release during the intestinal digestion of milk systems, but additional research is needed to fully comprehend lipid/protein interactions and their behavior inside the gastric and intestinal chambers.

#### Bioaccessibility of Curcumin

The bioaccessibility of curcumin was found to be significantly (*p* < 0.05) higher in the MHH digesta samples than the MMH and MLH digesta samples for all three selected timepoints (Figure 6C). These differences can be related to the microstructural changes occurring in the stomach, which influenced the nature of the curd fragments emptied in the digesta. Similar behavior was observed in our previous study, in which an acid milk gel, with softer gel fragments and an open microstructure, resulted in a significantly higher fraction of bioaccessible curcumin than a rennet gel (17). For all three recombined milk systems, there was no noticeable difference in the fraction of bioaccessible curcumin in the gastric digesta emptied at 20, 120, and 240 min. In our study, the high bioaccessibility of curcumin (>70%) for all recombined milk systems can be attributed to the fortification of curcumin as a nanoemulsion; because of its smaller droplet size and larger surface area, the incorporation of curcumin into the mixed micelles was enhanced (32, 33). These mixed micelles, having a hydrophilic surface with a nanometric size, are able to disperse in digestive fluids, boosting the likelihood of them passing through the mucus layers and reaching the intestinal epithelium for absorption (29, 34).

In contrast, when the curcumin masses in these digesta fractions were analyzed (Figure 6D), the gastric digesta emptied at 20 min showed a higher release of curcumin, which was followed by the gastric digesta emptied at 120 and 240 min. This is also in accordance with the concentration of oil droplets in the gastric digesta and the amount of FFA release. However, it is interesting to note that the fraction of curcumin in the MHH digesta samples showed a linear decreasing pattern whereas those in the MMH and MLH digesta samples followed a logarithmic decreasing trend. This logarithmic trend shows that the structural changes in MLH and MMH had a greater impact on the release of the curcumin fraction in the digesta than those in MHH. Moreover, positive correlations, i.e., MLH (*r* = 0.983), MMH (*r* = 0.999) and MHH (*r* = 0.983), between the concentration of curcumin recovered from the intestinal digesta and the amount of FFAs released were observed. This behavior has also been observed when carotenoids form part of oil-in-water emulsions (35), curcumin forms part of dairy gels (17) and capsaicinoids form part of whey protein emulsion gels (31).

## Conclusion

This work demonstrated the impact of the gastrointestinal digestion of recombined milks on the bioaccessibility of curcumin, highlighting the effect of the process-induced changes in the milks on the composition of the emptied gastric digesta. Both the nature of the preheat treatment used during SMP manufacture and the curcumin nanoemulsion supplementation modified the structure and consistency of the gastric curds. Under dynamic gastric conditions, the high-heat-treated milk proteins in MHH formed a loose and soft curd that resulted in faster outflow of the curd fragments along with entrapped curcumin nanoemulsions, compared with MLH and MMH. These differences in the gastric digesta profiles resulted in differences in the release of FFAs and the bioaccessibility of curcumin during intestinal digestion. In conclusion, the findings demonstrate the gastrointestinal bioaccessibility curcumin was better from MHH than from MLH and MMH and was dependent on the microstructural and compositional changes during the digestion of milk systems.

## Data Availability Statement

The original contributions presented in this study are included in the article/supplementary material, further inquiries can be directed to the corresponding author/s.

## Author Contributions

HQ: conceptualization, methodology, validation, investigation, formal analysis, data curation, writing—original draft, and visualization. AY: conceptualization, methodology, resources, writing—review and editing, project administration, supervision, and funding acquisition. AA-F: writing—review and editing and supervision. HS: conceptualization, writing—review and editing, and supervision. All authors contributed to the article and approved the submitted version.

## Conflict of Interest

The authors declare that the research was conducted in the absence of any commercial or financial relationships that could be construed as a potential conflict of interest.

## Publisher’s Note

All claims expressed in this article are solely those of the authors and do not necessarily represent those of their affiliated organizations, or those of the publisher, the editors and the reviewers. Any product that may be evaluated in this article, or claim that may be made by its manufacturer, is not guaranteed or endorsed by the publisher.
